# GlicoPro, Novel Standardized and Sterile Snail Mucus Extract for Multi-Modulative Ocular Formulations: New Perspective in Dry Eye Disease Management

**DOI:** 10.3390/pharmaceutics13122139

**Published:** 2021-12-13

**Authors:** Rita Mencucci, Giovanni Strazzabosco, Virginia Cristofori, Andrea Alogna, Daria Bortolotti, Roberta Gafà, Michela Cennamo, Eleonora Favuzza, Claudio Trapella, Valentina Gentili, Roberta Rizzo

**Affiliations:** 1 Eye Clinic, Careggi Hospital, Department of Neurosciences, Psychology, Pharmacology and Child Health (NEUROFARBA), University of Florence, 50134 Florence, Italy; michelacennamo@libero.it (M.C.); elefavuzza@gmail.com (E.F.); 2 Chemical, Pharmaceutical and Agricultural Sciences, Department of Chemical, University of Ferrara, 44121 Ferrara, Italy; giovanni.strazzabosco@unife.it (G.S.); virginia.cristofori@unife.it (V.C.); lgnndr1@unife.it (A.A.); brtdra@unife.it (D.B.); trpcld@unife.it (C.T.); valentina.gentili@unife.it (V.G.); 3 Department of Translational Medicine, University of Ferrara, 44121 Ferrara, Italy; gfr@unife.it; 4 Oncological and Medical Department, University Hospital of Ferrara Arcispedale Sant’Anna, 44124 Ferrara, Italy; 5 Laboratory for Technologies of Advanced Therapies (LTTA), University of Ferrara, 44121 Ferrara, Italy

**Keywords:** dry eye disease, snail mucus, opiorphin, artificial tears, corneal epithelium

## Abstract

This study aimed to evaluate the mucoadhesive and regenerative properties of a novel lubricating multimolecular ophthalmic solution (GlicoPro^®^) extracted from snail mucus and its potential anti-inflammatory and analgesic role in the management of dry eye disease (DED). GlicoPro bio-adhesive efficacy was assessed using a lectin-based assay, and its regenerative properties were studied in a human corneal epithelial cell line. In vitro DED was induced in human corneal tissues; the histology and mRNA expression of selected genes of inflammatory and corneal damage biomarkers were analyzed in DED tissues treated with GlicoPro. A higher percentage of bio-adhesivity was observed in corneal cells treated with GlicoPro than with sodium hyaluronate-based compounds. In the scratch test GlicoPro improved in vitro corneal wound healing. Histo-morphological analysis revealed restoration of cellular organization of the corneal epithelium, microvilli, and mucin network in DED corneal tissues treated with GlicoPro. A significant reduction in inflammatory and ocular damage biomarkers was observed. High-performance liquid chromatography-mass spectrometry analysis identified an endogenous opioid, opiorphin, in the peptide fraction of GlicoPro. In conclusion, GlicoPro induced regeneration and bio-adhesivity in corneal cells; moreover, considering its anti-inflammatory and analgesic properties, this novel ophthalmic lubricating solution may be an innovative approach for the management of DED.

## 1. Introduction

Dry eye disease (DED) is a multifactorial ocular disease affecting approximately 5–50% of the global adult population, with an increasing worldwide incidence [1]. DED symptoms, ranging from ocular discomfort to pain and vision disturbance, can become both physically and psychologically debilitating with a negative impact on a patient’s quality of life [2,3].

Etiological and risk factors that may play a pathogenic role in inducing DED include female sex, older age, Asian race, anterior and posterior blepharitis, environmental conditions, use of video display terminals, contact lens wearers, topical and systemic medications, autoimmune diseases, ocular surgery, and predisposing ocular anatomic abnormalities [4].

The pathophysiology of DED is related to alterations in ocular surface homeostasis, resulting in tear film instability, hyperosmolarity, ocular surface inflammation and damage, and neurosensory abnormalities [2]. Hyperosmotic or ocular surface desiccation stress that affects corneal and conjunctival epithelial cells induces ocular surface inflammation, which plays a crucial role in driving and maintaining DED. Both innate and adaptive immune cells are involved in inducing both subtypes of DED, namely evaporative dry eye disease and aqueous deficient dry eye disease. Regarding innate immune cell subsets on the ocular surface of eyes with DED, a significant reduction in the proportion of natural killer (NK) cells has been reported, suggesting that NK cells may play a protective role against DED pathology in the human ocular surface mucosa. However, cytofluorimetry analysis of the conjunctival cell population in patients with DED revealed an increased percentage of ocular surface CD14+ (monocytes/macrophages) and neutrophils, which is the effect of the proinflammatory cytokines and chemokines released by the conjunctival epithelial cells (e.g., TNF-α, IL-1α, IL-6, IL-8, GM-CSF, RANTES (CCL5), eotaxin-1 (CCL11), MCP1 (CCL2), and IP10 (CXCL10)) [5]. An additional mechanism of innate immune defense involves the secretion of human β-defensins (hBDs) and antimicrobial peptides active against several different bacteria, fungi, and enveloped viruses, which has been confirmed in vitro. The chemotactic effects of defensins on mast cells, T-cells, dendritic cells, and monocytes have led to the suggestion that defensins are a link between innate and adaptive immunity, thus providing an alternate mechanism of defense for the compromised ocular surface in DED [6].

Regarding the adaptive arm of the immune system involved in DED pathogenesis, an increased CD4/CD8 T lymphocyte ratio was observed in the outer layers of the conjunctival epithelium of patients with DED [5,7]. It is well known that hyperosmotic stress is a potent inflammatory stimulus in DED because it triggers the release of proinflammatory cytokines (interleukin [IL]-1a and b, IL-6) and chemokines (IL-8) by conjunctival epithelial cells, which drives a local inflammatory response, resulting in ocular tissue damage and ocular surface pain [8].

Moreover, the corneal apical surface and conjunctival epithelial cells are covered with a layer of glycocalyx that forms a boundary between these cells and the tear film. Membrane-tethered mucins are important components of the glycocalyx. Proinflammatory cytokines, including IL6, released from ocular surface epithelial cells in response to hyperosmolar stress, can modulate the expression of genes encoding membrane-tethered mucins (MUC1 and MUC4) [9].

Regarding the modulation of somatic neuropathic pain related to DED, pro-inflammatory cytokines such as IL-6 contribute to the activation of the sensory nerve terminals of trigeminal neurons, which innervate the ocular surface; moreover, chemokines (such as CXCL12 and CCL2) can reduce endogenous opioid–mediated analgesia. Indeed, endogenous opioids, such as pro-enkephalin, which are produced by immune cells and involved in controlling peripheral inflammatory pain with an analgesic action, are downregulated in DED8. One of the main endogenous metabolites is opiorphin, which is naturally present in biological human fluids (blood, urine, saliva, and other fluids). Opiorphin is also secreted in tears, where it prevents the degradation of encephalins, and thus inhibits pain perception [10,11]. Ozdogan et al. reported that opiorphin levels are increased in patients diagnosed with corneal foreign objects compared with healthy individuals, suggesting that opiorphin secretion increases in response to pain [12].

The complexity of DED pathogenesis has led to the development of several treatments that target different aspects of the disease. Artificial tears are typically considered the first-line treatment option in DED management along with anti-inflammatory therapy (topical corticosteroids, cyclosporine, and lifitegrast); eyelid hygiene and warm compresses are conservative measures in the case of blepharitis or in Meibomian gland dysfunction. Alternative therapies are targeted towards different mechanisms of DED simultaneously; in particular, there is interest in exploring the effect of naturally derived anti-inflammatory products and in addressing nerve abnormalities such as neurotrophic keratopathy or neuropathic pain (NP) [13,14,15]. The use of naturally derived actives might have fewer side effects and allow longer treatment.

It is already known that the active agents present in the *H. aspersa* mucus contribute to cell regeneration and reducing cell apoptosis, as demonstrated by in vitro studies on fibroblasts [16,17]. Moreover, venomous fish-hunting cone snails (Conus) produce potent non-opioid pain therapeutic peptides, suggesting a possible similar production by *H. aspersa* [18]. However, the benefit of *H. aspersa* mucus in the treatment of DED has not yet been investigated.

GlicoPro-based artificial tears (Lacricomplex^®^, FB Vision Spa, CE marked), are a multicomplex ocular formulation produced with a standardized technological extraction procedure for *H. aspersa* mucus. GlicoPro is characterized by the presence of mucopolysaccharides such as glycosaminoglycans (GAG) and sulfurated GAG (sGAG).

The purpose of this study was to evaluate the GlicoPro peptide signature and its effect on corneal epithelial cell repair and on experimentally induced DED human corneal cells. The analysis of a selected gene signature was performed on inflammatory biomarkers (IL-1β, IL-6, IL-8) and ocular damage markers (matrix metallopeptidase-9 (MMP9), MUC-4, and defensin β-2 (DEFB2)). Increased MMP9 expression has been observed in diseases that characterized the ocular epithelial surface and in the tear fluid of patients affected by DED [19,20,21]. The expression of MUC-4 was evaluated as it has clearing and lubricating functions that provides a barrier for corneal and conjunctival epithelia [22,23]. Human DEFB2 expression was analyzed as a marker of ocular surface damage in DED patients and its implication in antimicrobial protection [24,25].

## 2. Materials and Methods

### 2.1. GlicoPro Collection

The Helix aspersa snails were fostered in the private snail farming “Corte Frazza” (Ferrara, Italy). GlicoPro was collected by HelixPharma industries (Ferrara, Italy). The snail mucus was extracted for the purpose of this study using a patented extractor machine (Beatrix^®^; HelixPharma industries; Ferrara, Italy) [26], that collects about 600 mL of crude extract from 500 snails (about 10 kg) after 45 min. The mucus was obtained using low concentrations of NaCl (3%) that was sprayed on snails. The stress caused by this solution induced the snails to produce mucus, that was collected in underlying canisters. Then the snails were rehydrated and re-entered the field. The process does not cause mortality to the snails. Mucus was then sterilized with a peristaltic pump and a filtration device (0.2 µm; Pall, Milan, Italy) [27], specifically developed for mucus filtration and stored at 4 °C or −80 °C. The mucus is available by request to HelixPharma industries.

### 2.2. GlicoPro Characterization

GlicoPro was produced using a controlled and patented supply chain (Patent number FE102017000117547) and a patented method for the extraction (Patent number BO2011A000590) and filtration (0.2 μm; Pall, Milan, Italy). The characterization of GlicoPro was performed as previously reported [16].

#### 2.2.1. Qualitative and Quantitative Analysis of Specific Chemical Elements of GlicoPro

Allantoin and glycolic acid concentrations were simultaneously determined by HPLC analysis using a Beckman System Gold 125 instrument coupled with a Beckman 166 UV detector set at 200 nm and equipped with a Synergy Hydro-RP C-18 Column (250 mm × 4.6 mm I.D., 5 μm particle size Phenomenex, Torrance, CA, USA) [28]. The mobile phase was a potassium phosphate solution (KH_2_PO_4_, pH 2.9; 10 mM) and acetonitrile (CH_3_CN) with a flow of 0.7 mL/min at 30 °C. Elution was performed with isocratic of solution A (KH_2_PO_4_) for 10 min and reduced at 30% in 5 min. This percentage was maintained for 10 min for the perfect clean-up of the column. Then the percentage of solution A was restored at 100% in 10 min and maintained for another 10 min for the reconditioning of the column before injecting the next sample. The standard curve was prepared in triplicate dissolving increasing amounts of allantoin (Fluka, Milan, Italy) and glycolic acid (Sigma-Aldrich, Milan, Italy) in phosphate buffer.

Spectrophotometric assay was used to quantify sulfated and non-sulfated glycosaminoglycans (GAGs). The content of sulfated GAGs was determined using the DMMB (1,9-Dimethyl-Methylene Blue, Sigma-Aldrich) assay according to the manufacturer’s instructions. Standards for GAGs quantification (chondroitin-sulfate, heparan-sulfate, and dermatan-sulfate, Sigma-Aldrich, Milan, Italy) were prepared in 100 mM ammonium acetate in a range of concentrations 0–50 µg/mL. The measure was performed in duplicate in a microplate reader (Infinite 200 PRO Series Multimode Reader from Tecan Trading AG; Tecan, Männedorf, Switzerland) at 525 nm.

The amount of total phenols, generally related to the antioxidant activity of a sample, was measured with the Folin-Ciocalteou (F-C) assay. Briefly, 0.5 g of the standard polyphenol gallic acid (Sigma Aldrich, Burlington, MO, USA) were dissolved in 10% ethanol. In a cuvette 20 µL of the sample were mixed with 1.58 mL of deionized water, 100 µL of F-C reagent, and 300 µL of 20% Na_2_CO_3_ solution. The analysis was performed after 90 min in a dark field. The total phenolic content was calculated from the calibration curve, obtained with increasing concentrations of gallic acid and measured with a spectrophotometer (Beckman DU 520, Beckman, Milan, Italy) at 765 nm.

Free sugars (D-glucose and D-fructose) and disaccharides (sucrose) were analyzed with the D-glucose D-fructose Kit (Megazyme Inc., Chicago, IL, USA). D-glucose concentration was determined before and after sucrose hydrolysis with the β-fructosidase enzyme. The D-fructose amount was achieved as consequence of D-glucose quantification, after isomerization caused by the phosphoglucose isomerase.

Quantification of collagen was performed using a colorimetric method for hydroxyproline analysis. Briefly, a standard curve with increasing amount of hydroxyproline was prepared. Mucus sample was dissolved in 100 µL of concentrated hydrochloric acid for the release of the hydroxyproline aminoacid after complete protein hydrolysis. Hydroxyproline was quantified using the oxidation buffer citrate/acetate and DMAB.

#### 2.2.2. LC/MS Analysis of GlicoPro

GlicoPro was dissolved in 1.5 mL of a 60% acetonitrile, 40% water, and 0.1% formic acid solution and filtered off to a 0.22 μm regenerate cellulose filter. The clear solution was then analyzed with ESI-Q-TOF Nano HPLC-CHIP Cube Agilent 6520 instrument (Agilent Technologies, Santa Clara, CA, USA) using a linear gradient (0.4 µL/min) from 0% solvent A (97% water/3% acetonitrile/0.1% formic acid) to 80% solvent B (97% acetonitrile/3% water/0.1% formic acid) in 10 min and from 80% to 5% solvent B in 5 min using a Zorbax C18 Column (43 mm × 75 µm, 5 µm) equipped with an enrichment column (4 mm, 40 nL).

#### 2.2.3. Microbiological Characterization

To test the eventual microbiological contamination, 100 μL of GlicoPro were plated on culture dishes containing culture media Tryptic Soy agar (TSA) (Biomerieux, Grassina, Italy). The number of colonies was evaluated after 24–48 h at 37 °C and expressed as colony forming unit (CFU).

The identification of contaminating bacteria was performed by Gram staining (Liofilchem, Abruzzi, Italy). The presence of contaminating fungi was evaluated by plating GlicoPro on Sabouraud medium plates (Biomerieux, Grassina, Italy).

### 2.3. GlicoPro Bio-Adhesivity Test

We used an easy and standardized protocol for the evaluation of GlicoPro bio-adhesive properties using a lectin-based assay [29]. Lectine is known to bind to mucines expressed on mucosal surfaces. Therefore, this assay tested the ability of substances to bind the mucosa and consequently interfere with lectin-mucin interactions. Briefly, human corneal epithelial cell line (HCE-2) (ATCC, Manassas, VA, USA; Number CRL-11135) were seeded in eight-chamber slides at a final concentration of 20,000/well, obtaining 80% confluence. After 24 h at 37 °C and 5% CO_2_, the keratinocyte serum-free medium (Gibco; Merck Life Science S.r.l., Milan, Italy; Number: 17005-042) supplemented with 0.05 mg/mL bovine pituitary extract (Gibco), 5 ng/mL epidermal growth factor, 500 ng/mL hydrocortisone, and 0.005 mg/mL insulin (Gibco) was removed and the cells were allowed to dry for 15 min. Cells were fixed with 100 µL of 1:1 methanol: acetone solution for 30 min at −20 °C and allowed to dry again at 22 ± 2 °C. The cells were finally rehydrated for 5 min by adding 500 µL of phosphate-buffered saline (PBS). Next, 200 µL of GlicoPro was added to the cells. Further, 200 µL of PBS was used as a negative control and 200 µL of a bio-adhesive solution containing 1 g/10 mL of Sucralfate gel, diluted 1:5 in sterile water and a solution of 0.8 mg/mL of a natural molecular complex containing polysaccharides, natural mineral, and Arabic gum was used as a positive control. After 15 min at 37 °C, 200 µL of biotinylated Lectine 10 µg/mL was added to each well and the wells were incubated for 30 min at 37 °C. Each well was then washed three times with 500 µL of 0.05% tween20-PBS for 5 min with agitation. After the washes, we added 200 µL of Streptavidin-HRP (2.5 µg/mL) diluted 1:100 for 1 h at 37 °C. Cells were then washed again in triplicate, and 100 µL of TMB substrate was added to each well for 5 min. The reaction was stopped with 100 µL of HCl 1N and 100 µL was transferred to a 96-well plate and read at 450 nm. The absorbance is inversely correlated with the bio-adhesive properties of the tested substance. Each experiment was performed in triplicate.

### 2.4. Scratch Test

The scratch test was used to assess the ability of GlicoPro to regenerate damaged cells. The human corneal epithelial cell line (HCE-2) (ATCC, Manassas, VA, USA; Number CRL-11135) was cultured in keratinocyte serum-free medium (Gibco; Number: 17005-042) supplemented with 0.05 mg/mL bovine pituitary extract (Gibco), 5 ng/mL epidermal growth factor, 500 ng/mL hydrocortisone, and 0.005 mg/mL insulin (Gibco) at 37 °C and 5% CO_2_. The cells were cultured in a culture-insert 2 well (Ibidi GmbH, Gräfelfing, Germany) to obtain a scratch. After 24 h, the cell reached a 90% confluence and the inserts were removed, and 100–500 µL of GlicoPro was added to the test sample. Images of the wounded areas were captured before the addition of GlicoPro and after 24 h of treatment. The control group received 100–500 µL PBS (PBS; Gibco). The wound area was assessed at 0 and 24 h with pixel areas using Adobe Photoshop Elements 2020 (Adobe, San Jose, CA, USA). The treated and control samples were compared. Each experiment was performed in triplicate.

### 2.5. Experimentally Induced In Vitro Dry Eye in Human Corneal Tissues

Corneal tissues were donated for research purposes from the Eye Biobank: Fondazione Banca degli Occhi del Veneto, Italy. Each specimen was deemed unsuitable for transplantation because of failure of serologic screening. These specimens were donated to research after informed consent. All tissues were <7 days postmortem. Corneal tissues were cut from the eyes with a scalpel, and a section of about 3 mm of sclera was maintained to preserve the corneal shape and to facilitate the subsequent culture. Tissues were maintained in CorneaMax ^®^ fluid, suitable for organ’s culture. We used human corneal tissues as they present all the metabolic, anatomical, and functional features of the living organ. As previously reported, 30 min prior to experiments, all corneal tissues were maintained to a temperature of 32 °C, which corresponds to the temperature of human corneal surface. Then, corneal tissues were placed for 24 h at <40% relative humidity, 40 ± 5 °C temperature and 5% CO_2_ to mimic dryness [6,30,31].

The corneal tissues were then treated with GlicoPro 100 µL/day. A similar culture was treated with PBS 100 µL/day to serve as a control. Cell viability was assessed by 0.4% trypan blue solution (Sigma Chemical Co., St. Louis, MO, USA) for 1 min. Morphology, histology, and mRNA expression of selected genes at 24 h post-treatment were assessed on corneal tissues.

### 2.6. Histological Analysis

At the end of GlicoPro treatment, the corneal tissues were fixed with 10% formalin solution (HT501128), cut in vertical sections (4 μm thick) with a microtome and hematoxylin, and eosin stained. Light microscope was used to analyze histological samples, assessing epithelial morphology.

### 2.7. Electron Microscope Analysis

First, 2.5% glutaraldehyde in 0.1 M phosphate buffer was used to fix corneal tissues for 2 h at 4 °C. After three washes for 5 min with 0.1 M phosphate buffer, the tissues were placed in 1% OsO4 in 0.1 M phosphate buffer, then dehydrated with a graded series of ethanol and of hexamethyldisilane. The specimens were mounted on aluminum stubs with silver-conducting paint, coated with a gold sputter coater, Quorum Q 150R S, and observed under a scanning electron microscope (Zeiss Evo 40, Zeiss, Oberkochen, Germany).

### 2.8. Transcriptional Study of mRNA

mRNA was obtained from the corneal tissues using the RNeasy Mini Kit (Qiagen, Hilden, Germany), according to the manufacturer’s protocol. cDNA was synthesized using a SuperScript kit (Thermo Fisher, Waltham, MA, USA). Real-time polymerase chain reaction (PCR) was performed in triplicate in a final reaction volume of 25 μL using the ABI PRISM 7500 Real Time PCR System (Applied Biosystems, Waltham, MA, USA) with a TaqMan^®^ assay (Ambion-Applied Biosystems, Thermo Fisher, Waltham, MA, USA). cDNA amplification was obtained using TaqMan Universal PCR Master Mix and TaqMan gene expression assay provided as a 20× Assay mix (Human Glycerolaldehyde 3-Phosphate Dehydrogenase (GAPDH) as the calibrator gene: TaqMan probe GAPDH Hs99999905_m1; Human MMP9: TaqMan probe MMP9 Hs00234579_m1; HumanMUC4: TaqMan probe MUC4 Hs00366414_m1; HumanDEFB2 (Human beta-defensin-2): TaqMan probe DEFB2 Hs 00175474_m1) (human IL-1β: TaqMan probe IL-1β Hs01555410_m1; Human IL-6: TaqMan probe IL-6 Hs00174131_m1; Human IL-8: TaqMan probe IL-8 Hs00174103_m1; Human IL-1α: TaqMan probe IL-1α Hs00174092_m1; Human TNF-α: TaqMan probe TNF-α Hs02621508_s1; Human IL-10: TaqMan probe IL-10 Hs00961622_m1). The PCR conditions were: 95 °C for 10 min (AmpliTaq Gold DNA Polymerase activation, Thermo Fisher, Waltham, MA, USA) followed by 40 amplification cycles (95 °C for 15 s, then 60 °C for 1 min). Relative gene expression was calculated using the 2 (-Delta C[T]) method.

### 2.9. GlicoPro Peptide Analysis

High-performance liquid chromatography-mass spectrometry (HPLC-MS) analysis was performed with a HPLC-ESI/HRMS QExactive (Thermo Scientific, Milan, Italy) using a 0.5 mL gradient of 2–28% acetonitrile in water containing 0.1% formic acid in 10 min on a Hipersyl Gold C18 column (100 × 2.1 mm, 1.9 mm) at 30 °C. The MS spectra were acquired in ESI positive full MS 200–2000 Th at 70 k FWHM (at 200 *m*/*z*) and the Top4 Data Dependent MS/MS at 17.5 a resolution. The primary sequence of the peptide was analyzed using Peptide2Protein software (http://www.pinet-server.org/pinet/peptideToProtein (accessed on 10 September 2021)) to map peptides into proteins using a fast peptide-to-protein matching.

### 2.10. Statistical Analysis

Statistical comparisons of the data were performed using the Student’s *t*-test as the all the data displayed a normal distribution based on the Kolmogorov–Smirnov and D’Agustino-Pearson normality tests. Differences were considered significant at *p* < 0.05. Statistical analyses were performed using the GraphPad Prism version 8 (GraphPad Software, San Diego, CA, USA).

## 3. Results

### 3.1. GlicoPro Characterization

The patented extraction and the specific analysis reported in Materials and Methods section allowed the standardization of the GlicoPro, that presented a specific characterization, as reported in Table 1. We observed a high prevalence of mucopolysaccharides, such as GAG and sGAG, which might enhance the bio-adhesivity of GlicoPro.

### 3.2. Effects of GlicoPro on HCE-2 Cell Growth

**Figure 1:**
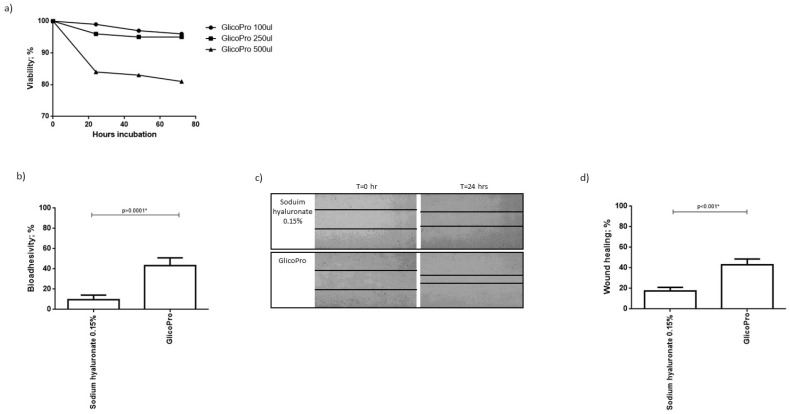
GlicoPro effect on HCE-2 cells. (**a**) Effect of different doses (100–500 µL) of GlicoPro on the proliferation of HCE-2 cells after 72 h of culture. (**b**) The bio-adhesivity of GlicoPro compared with sodium hyaluronate 0.15%. Each experiment was performed in triplicate. (**c**,**d**) Scratch assay: representative results in the HCE-2 cell line after 24 h of treatment with GlicoPro or sodium hyaluronate 0.15%. Each experiment was performed in triplicate. The data are presented as mean ± standard deviation. * significative *p* value.

We analyzed the proliferation of HCE-2 cells, cultured in a culture-insert 2 well to obtain a scratch, in the presence of different concentrations of GlicoPro. After 72  h of culture, 100–250 µL of GlicoPro did not affect cell viability (viability: 95 ± 5%). As can be seen in Figure 1a, 500 µL was slightly cytotoxic (viability: 82 ± 1%). As a result, we assessed the effect of 100 µL of GlicoPro.

### 3.3. Bio-Adhesivity

Bio-adhesion was evaluated by lectin binding of surface mucins using biotin-streptavidin. The decrease in absorbance is proportional to the interaction between the tested substances and the mucosal cells. The mucoadhesive ability is reported as a percentage of inhibition of glycoprotein-lectin binding, which corresponds to the extent of mucosal sites interacting with GlicoPro. The assay was conducted on HCE-2 cells, and GlicoPro was compared to sodium hyaluronate 0.15%. We observed that the percentage of bio-adhesivity was significantly higher in cells treated with GlicoPro than in cells treated with sodium hyaluronate-based compound (*p* < 0.0001; Student’s *t*-test) (Figure 1b).

### 3.4. Scratch Test

GlicoPro treatment induced a faster reconstitution of the scratch in the HCE-2 cell monolayer compared to the sodium hyaluronate 0.15% treated samples (*p* < 0.001; Student’s *t*-test) (Figure 1c,d).

### 3.5. Dry Eye Assay

**Figure 2:**
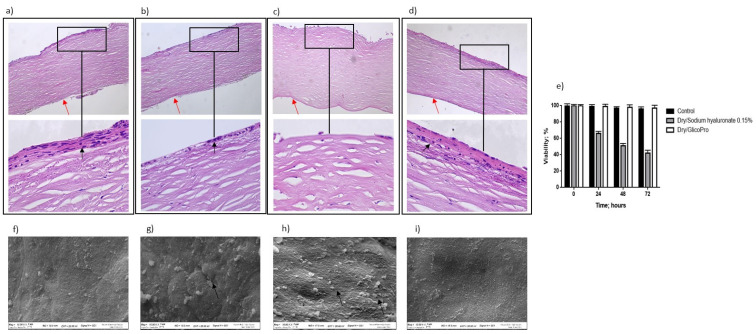
Histo-morphological analysis. Histo-morphological analysis of (**a**) control group corneal tissues; (**b**) dry eye; (**c**) control dry eye (100 µL sodium hyaluronate 0.15%), and (**d**) dry eye corneal tissues treated with GlicoPro for 24 h. Magnifications: 10× (upper panels), 60× (lower panel). Red arrows indicated endothelial cell layer; black arrows indicated stratified squamous epithelium. (**e**) Cell viability assessed by trypan blue staining. Scanning electron microscopy images of human corneal tissue in (**f**) control; (**g**) dry eye; (**h**) control dry eye (100 µL sodium hyaluronate 0.15%); and (**i**) GlicoPro-treated dry eye conditions. Each condition was analyzed in triplicate. Scale bars = 100 μm.

The ability of GlicoPro to prevent the inflammatory status typical of DED was determined. Figure 2 shows the overall morphology of the control corneal tissue, control dry eye (100 µL sodium hyaluronate 0.15%), and dry eye treated with GlicoPro (100 µL GlicoPro) for 24 h, as observed under a light microscope. The tissue morphology of the control was preserved (Figure 2a), with stratified squamous epithelium, thin acellular Bowman’s membrane (black arrow), stroma with collagen fibers and thin fibroblasts, and simple squamous to cuboidal endothelium (red arrow). Twenty-four hours after dry eye induction, an enhanced loss of cellular organization of the stratified squamous epithelium was observed (Figure 2b, black arrow). A remarkable loss of cellular organization of the stratified squamous epithelium was observed in the dry eye condition treated for 24 h with sodium hyaluronate 0.15% compared to the control (Figure 2c). Notably, the stratified squamous epithelium in the DED corneal tissues was regenerated by 24 h GlicoPro treatment (Figure 2d, black arrow). Even if all the conditions presented viable cells, as assessed by trypan blue staining (Figure 2e), the DED corneal tissues showed a loss of cellular organization, related to the loss of water due to severe dry experimental conditions. Therefore, high temperature and low humidity could reproduce the dry environmental conditions that cause ocular discomfort and inflammation. GlicoPro treatment was able to induce the regeneration of the stratified squamous epithelium, showing a clear restoration of the cellular organization within 24 h of treatment (Figure 2d, black arrow).

### 3.6. Corneal Tissue Analysis Using Scanning Electron Microscopy

Scanning electron microscopy analysis revealed that the control tissue had no obvious superficial epithelial sloughing, or increased intercellular gaps (Figure 2f). A significant increase in sloughing of superficial epithelial cells was observed in dry eye (Figure 2g, black arrow) and control dry eye condition (100 µL sodium hyaluronate 0.15%) (Figure 2h, black arrows). In dry eye condition the treatment with GlicoPro for 24 h, showed a clear restoration of the superficial epithelial integrity (Figure 2i).

### 3.7. Quantification of Inflammation and Ocular Damage Biomarker Expression

**Figure 3:**
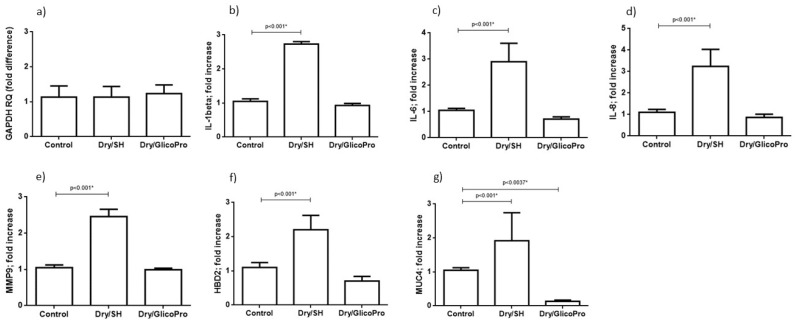
Quantitative reverse transcription polymerase chain reaction. Quantitative reverse transcription polymerase chain reaction results of (**a**) GAPDH, (**b**) interleukin (IL)-1 beta, (**c**) IL-6, (**d**) IL-8, and (**e**) matrix metallopeptidase-9 (MMP9), (**f**) defensin β-2 (DEFB2), (**g**) mucin (MUC)4 levels in corneal tissues under normal conditions (37 °C) and dry eye conditions (Dry) wit sodium hyaluronate 0.15% (SH) or with GlicoPro treatments. The results are expressed as the mean of triplicate experiments after 24 h of treatment. The data are presented as mean ± standard deviation. * significative *p* value.

The corneal tissues were harvested and analyzed for the expression of inflammatory markers (IL-1B, IL-1A, IL-6, IL-8, TNF-A, IL-10) and ocular damage markers (MMP9, MUC-4, and HBD2). qRT–PCR results confirmed the cellular viability in all culture conditions. In fact, the expression of housekeeping gene GAPDH was comparable in all experimental settings (Figure 3a). qRT–PCR revealed increased inflammatory IL-1β, IL-6, IL-8 expression at 24 h following the induction of dry-eye and 24 h treatment with 100 µL sodium hyaluronate 0.15% (SH) in comparison with the control corneal tissue (*p* < 0.001; Student’s *t*-test) (Figure 3b–d). Treatment with GlicoPro reconstituted the basal levels of these inflammatory cytokines (Figure 3b–d). IL-1α, TNF-α and Il-10 were not modified by dry-eye condition in comparison with the basal level.

Overexpression of MMP9, MUC4, and HBD2 was observed at 24 h following the treatment with 100 µL sodium hyaluronate 0.15% (SH) of dry eye condition in comparison with the control corneal tissue (*p* < 0.001; Student’s *t*-test) (Figure 3e–g). The treatment with GlicoPro reconstituted the basal levels of all ocular damage biomarkers tested (Figure 3e–g). Interestingly, GlicoPro treatment reduced MUC4 expression in comparison with control basal condition (*p* = 0.037; Student *t* test) (Figure 3g). The other variables did not differ between GlicoPro treatment and control basal condition (IL-1β *p* = 0.2; IL-6 *p* = 0.057; IL-8 *p* = 0.25; MMP9 *p* = 0.42; HBD2 *p* = 0.10; Student’s *t*-test).

### 3.8. Opiorphin Detection in GlicoPro

**Figure 4:**
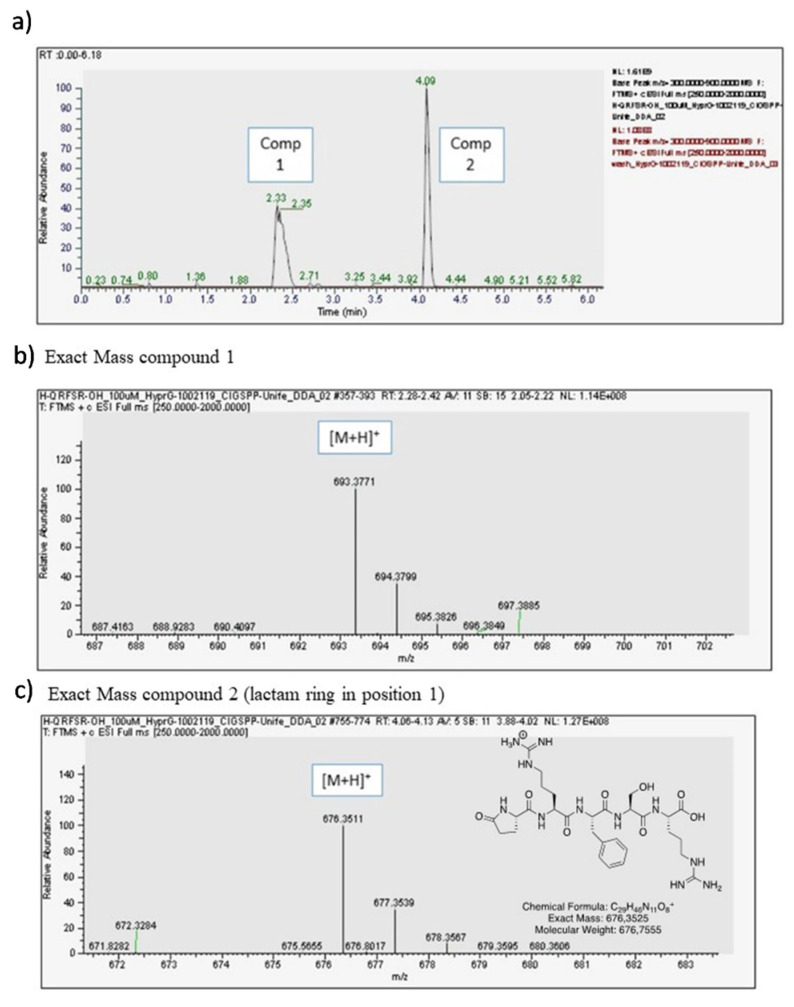
Opiorphin detection in GlicoPro. (**a**) High-performance liquid chromatography chromatogram of GlicoPro; (**b**) exact mass of compound 1, identified as opiorphin; (**c**) exact mass of compound 2, identified as lactame derivative of opiorphin in position 1.

GlicoPro is characterized by the presence of a complex mixture of a variety of biological molecules, such as high and low molecular weight proteins, glycosaminoglycans, minerals, and other secondary metabolites (Table 1). The most interesting molecules present in GlicoPro are small peptides (<5 kDa molecular weight). During our efforts to characterize the GlicoPro components, we selected the peptide fraction below 3 kDa for identification of these peptides via HPLC-MS, as representative of a class of biologically active peptides. The complex MS spectra clearly showed one peptide with a mass/charge ratio of 693.04 Da (data not shown). The high-resolution Q-trap analysis indicated the presence of two compounds, corresponding to a compound with an exact mass of 693.3771 uma (Compound 1) and a compound with an exact mass of 676.3511 uma (Compound 2) (Figure 4a). MS/MS analysis confirmed the sequence of the peptide as H-Gln-Arg-Phe-Ser-Arg-OH related to the first peak of the chromatogram (Figure 4b), whereas the second was the corresponding peptide, where the first glutamine residue was cyclized to form a lactam ring (Figure 4c). The sequence was analyzed and identified as human opiorphin. The concentration, as determined by MS/MS analysis, was 1500 pg/mL.

## 4. Discussion

In this study, we have described the biochemical profile of a novel topical ophthalmic formulation, GlicoPro, consisting of a multicomplex of mucopolysaccharides developed as lubricating eye drops in the management of DED (Lacricomplex^®^). Moreover, we tested the rheological and immunomodulating properties and cytoprotective effects of GlicoPro formulations using in vitro studies and an experimentally induced in vitro dry eye human corneal tissue [6,30,31]. A previous experimental study testing the mucus bio-adhesive properties of *H. aspersa* on human keratinocytes demonstrated that the presence of mucopolysaccharides improved the adhesion of mucus to the skin, because hydrogen bonding of adjacent water molecules and the stimulation of endogenous hyaluronate synthesis resulted in an increase in water-binding capacity and viscoelasticity of the skin [17].

Regarding the management of DED symptoms, hyaluronic acid is the most common GAG used as an artificial tear because of its excellent water-holding capacity, molecular weight, and viscosity, which enhance ocular surface hydration and minimize friction [32]. The presence of sulfurated mucopolysaccharide extracted from *H. aspersa* mucus provides a desirable mucoadhesive strength for the GlicoPro ocular formulation, as demonstrated by the higher percentage of bio-adhesivity in HCE-2 cells treated with GlicoPro than with other common hyaluronic acids. Optimal topical retention is necessary for clinical activity and effectiveness.

In addition, the properties of natural *H. aspersa* muller mucus that induce cell proliferation and migration have already been amply demonstrated in in vitro studies on fibroblasts and keratinocytes, suggesting that snail mucus is able to improve the in vitro wound healing process [16,17]. Our results confirm the ability of the GlicoPro ocular formulation extracted from snail mucus to improve in vitro corneal wound healing, as demonstrated by the scratch assay on HCE-2 cells, showing possible beneficial effects for the treatment of DED-associated corneal micro-erosions.

We have described an in vitro human corneal tissue culture exposed to dry environmental cultivation conditions that simulate many of the DED characteristics, primarily inflammation [6,30,31]. Our transcription analysis of the gene signature for epithelial dryness in corneal tissue confirmed previous clinical findings showing increased production of proinflammatory cytokine expression levels (IL-6, 8, 1β) during stressed dry eye conditions [6,33]. These results also indicated that GlicoPro has direct anti-inflammatory effects when used in an experimental model of DED, as it reconstituted the basal levels of the inflammatory cytokines tested (IL-6, 8, 1β). Moreover, the proinflammatory cytokines activated by the stressed ocular surface in DED have been demonstrated to increase enzymes that can digest glycocalyx, such as MMP9, along with mucins, containing side-chain structural and chemical components to form a network, resulting in glycocalyx damage.

The superficial corneal and conjunctival epithelia express membrane-associated mucins (MUCs 1, 4, and 16) that form a dense glycocalyx at the epithelial-tear film interface act as a lubricating agent and provide a barrier function to the epithelia. Upregulation of MUC4 mRNA has been shown to occur in an in vitro DED model, suggesting that this mucin is an early biomarker of ocular surface damage. MUC4 overexpression acts as a positive signal to stimulate the production of mucins in stressed situations in which mucin protein is absent [6]. In line with previous findings, our results revealed a significant increase in the expression of MUC4 and MMP-9 in corneal tissue exposed to the dry eye condition, suggesting that cytokine-mediated increases in the levels of MUC4 and MMP-9 could represent a physiological response to enhance the ocular surface defense against microbial stress [9].

Therefore, HBD-2 not only has chemotactic action on immune cell subpopulations of the innate and adaptive arms, but by stimulating histamine release from conjunctival mast cells may induce signs and symptoms of ocular irritation. Increased expression of HBD-2 may offer protection against antimicrobial activity, but may also be involved in ocular surface damage, as observed in subjects with DED, whose increased levels of HBD-2 in tears were considered to be a sign of ocular irritation [6]. Our data confirm that increases in MUC4, MMP9, proinflammatory cytokine-mediated, and higher levels of HBD-2 in DED may partially contribute to ocular surface damage in conditions associated with ocular surface inflammation. The use of topical GlicoPro in an experimentally induced human corneal tissue model showed a significant reduction in MMP-9, MUC-4, and HBD-2 expression, suggesting its potential protective role in mitigating the inflammatory damage caused by DED on the human ocular surface. Interestingly, MUC4 levels were lower in GlicoPro treated sample in comparison with control. This is a clear sign of ameliorated condition, that contrast any stress present in an in vitro condition, that might be present also in the control sample.

Histopathological analysis confirmed a detrimental effect on the corneal epithelial cells when exposed to dry eye conditions, with a disruption of epithelial integrity and a disorganization of epithelial stratification. The regeneration of the stratified squamous epithelium and the restoration of the superficial epithelial integrity, as detected by scanning electron microscopy, after treatment with GlicoPro indicate that the use of a formulation containing multicomplex mucopolysaccharides interacting with the mucus and aqueous layers of the tear film prevents and/or reduces the molecular and structural modifications typical of DED at the corneal epithelium level.

Finally, another important pathogenetic aspect to consider in the management of DED is neurosensory abnormalities. Inflammation of the ocular surface can result in damage to the corneal sub-basal nerve plexus, which detects mechanical, thermal, and chemical stimuli, and generates output perceivable as pain or a range of dysesthesias [34]. Our results indicated the presence of opiorphin in the protein supply of GlicoPro at the same concentration that is normally present in tears [12]. This peptide, which acts as a natural potent non-opioid endogenous analgesic, may have therapeutic effects on the neurosensory abnormalities responsible for DED symptoms such as NP.

To summarize, the present study has demonstrated the in vitro anti-inflammatory action; optimal mucoadhesive, regenerative properties; and potential analgesic role of the GlicoPro ocular formulation. Thus, GlicoPro could be a novel multitarget product to address the different pathogenetic aspects of DED. Further clinical evaluation is necessary to determine whether this formulation can effectively counteract the pathological effects of DED in human patients.

## Figures and Tables

**Table 1:** Characterization of GlicoPro.

Specifications	Values	Unit of Measure
Appearance	Clear	
Color	Light yellow	
Odor	Odorless	
Solubility in water	Soluble	
Solubility in organic solvents	Insoluble	
pH	7/8	
Density	1/1.1	
Moisture	1/3	g/L
Dry matter	0.1/0.2	%
Elements	250/350	mg/L
Heavy metals	Absent	
Proteins	90/200	mg/L
GAGs (sulfated)	29/90	mg/L
Non-sulfated GAGs (hyaluronic acid)	70/80	mg/L
Glycolic acid	<200	mg/L
Allantoin	<20	mg/L
Total polyphenols	70/80	mg/L
Total sugars	10/27	mg/L
Total microbial load	Absent	cfu/mL
Pesticides	Absent	
Preservatives	Absent	
Endotoxins	<0.25	EU/mL

## Data Availability

All data available are reported in the article.
